# Serum insulin-like growth factor-I in diabetic retinopathy

**Published:** 2011-08-27

**Authors:** John F. Payne, Vin Tangpricha, Julia Cleveland, Michael J. Lynn, Robin Ray, Sunil K. Srivastava

**Affiliations:** 1 Department of Vitreoretinal Surgery and Disease, Emory University, Atlanta, GA; 2 Division of Endocrinology, Metabolism & Lipids, Department of Medicine, Emory University, Atlanta, GA; Staff Physician, Atlanta VA Medical Center, Decatur, GA; 3 Department of Biostatistics and Bioinformatics, Emory University, Atlanta, GA; 4 Department of Vitreoretinal Surgery and Disease, Cole Eye Institute, Cleveland, OH

## Abstract

**Purpose:**

To assess the relationship between serum insulin-like growth factor I (IGF-I) and diabetic retinopathy.

**Methods:**

This was a clinic-based cross-sectional study conducted at the Emory Eye Center. A total of 225 subjects were classified into four groups, based on diabetes status and retinopathy findings: no diabetes mellitus (no DM; n=99), diabetes with no background diabetic retinopathy (no BDR; n=42), nonproliferative diabetic retinopathy (NPDR; n=41), and proliferative diabetic retinopathy (PDR; n=43). Key exclusion criteria included type 1 diabetes and disorders that affect serum IGF-I levels, such as acromegaly. Subjects underwent dilated fundoscopic examination and were tested for hemoglobin A1c, serum creatinine, and serum IGF-I, between December 2009 and March 2010. Serum IGF-I levels were measured using an immunoassay that was calibrated against an international standard.

**Results:**

Between the groups, there were no statistical differences with regards to age, race, or sex. Overall, diabetic subjects had similar serum IGF-I concentrations compared to nondiabetic subjects (117.6 µg/l versus 122.0 µg/l; p=0.497). There was no significant difference between serum IGF-I levels among the study groups (no DM=122.0 µg/l, no BDR=115.4 µg/l, NPDR=118.3 µg/l, PDR=119.1 µg/l; p=0.897). Among the diabetic groups, the mean IGF-I concentration was similar between insulin-dependent and non-insulin-dependent subjects (116.8 µg/l versus 118.2 µg/l; p=0.876). The univariate analysis of the IGF-I levels demonstrated statistical significance in regard to age (p=0.002, r=-0.20), body mass index (p=0.008, r=−0.18), and race (p=0.040).

**Conclusions:**

There was no association between serum IGF-I concentrations and diabetic retinopathy in this large cross-sectional study.

## Introduction

Diabetes mellitus continues to be a tremendous health burden throughout the world. The molecular pathophysiology of diabetic retinopathy, which remains the leading cause of blindness in Americans aged 20 to 74 years of age, is complex and involves multiple mechanisms [1]. Retinal neovascularization is a major cause of sight-threatening complications in diabetic patients, and the mechanism of its development is not completely understood. Experimental studies performed over 40 years ago demonstrated that pituitary ablation resulted in remission of diabetic retinopathy, possibly because of reduced circulating levels of growth hormone [2–4]. However, additional studies led investigators to suggest that a reduction in secondary growth factors, such as insulin-like growth factor-I (IGF-I), caused the remission of retinopathy [5,6].

IGF-I, or somatomedin C, is homologous to proinsulin, and is the major mediator of the growth-promoting effects of growth hormone [7]. While experimental and clinical evidence suggests that serum IGF-I concentrations may be involved in the development of diabetic retinopathy, the relationship is still controversial. Several studies have reported that higher serum IGF-I levels may be a risk factor for the development of severe diabetic retinopathy [7–9]. Conversely, a few studies have shown no association between serum IGF-I levels and the development or progression of diabetic retinopathy [10–13]. It is possible that this disagreement stems from the various assays used to measure IGF-I levels. The purpose of this study was to assess the relationship between serum IGF-I levels and diabetic retinopathy, using a novel immunoassay calibrated to the new World Health Organization standard.

## Methods

### Study design

The Emory University Institutional Review Board approved this study, which was conducted in accordance with the Health Insurance Portability and Accountability Act regulations. A clinic-based cross-sectional study was designed at the Emory Eye Center, and all patients were enrolled between December 16, 2009 and March 21, 2010. Patients who were seen in the retina, glaucoma, cornea, and comprehensive ophthalmology clinics during the enrollment period were considered potential study subjects. These patients were screened by the study investigators to determine their age, race, sex, and diabetes status. After undergoing routine ophthalmic examination, which included dilated fundoscopy, subjects were recruited for inclusion in the four study groups. Attempts were made to keep the study groups equally matched according to age, race, and sex.

### Study subjects

Subjects were divided into four distinct groups, based on their diabetes status and retinopathy findings. The first group consisted of subjects without diabetes. Subjects in this group were not excluded if they had other forms of ocular disease, such as uveitis or macular degeneration. The no background diabetic retinopathy (no BDR) group consisted of subjects with type 2 diabetes but no evidence of diabetic retinopathy, such as microaneurysms, cotton-wool spots, intraretinal hemorrhages, or macular edema. Subjects in the nonproliferative diabetic retinopathy (NPDR) group had evidence of retinopathy, such as microaneurysms, cotton-wool spots, intraretinal hemorrhages, or macular edema, but no evidence of retinal or iris neovascularization. The proliferative diabetic retinopathy (PDR) group consisted of subjects with neovascularization on the optic disc, retina, or iris, with or without vitreous hemorrhage or prior panretinal photocoagulation. When the diabetic retinopathy was asymmetric, the subject was assigned to the group corresponding to the eye with the worse retinopathy findings.

Subjects were excluded if they had type 1 diabetes, were younger than 18, or were older than 90 years of age. Subjects with prior diseases that suggested baseline alterations in IGF-I levels, such as acromegaly, were also excluded. Patients who were cognitively impaired or unable to provide written informed consent were excluded as well.

### Biochemical measurements

Once enrolled in the study, subjects completed a medical history questionnaire and had their blood drawn. Patients were not required to be fasting for this blood draw. One blood sample was immediately tested for hemoglobin A1c. Other whole blood, serum, and plasma samples were frozen at −80 °C. Serum samples were later tested for creatinine (Beckman Coulter, Inc.; Brea, CA), 25-hydroxyvitamin D (Immunodiagnostic Systems; Scottsdale, AZ), and IGF-I levels (Immunodiagnostic Systems; Scottsdale, AZ). The results of the 25-hydroxyvitamin D testing were reported separately (data not shown).

**Figure 1:**
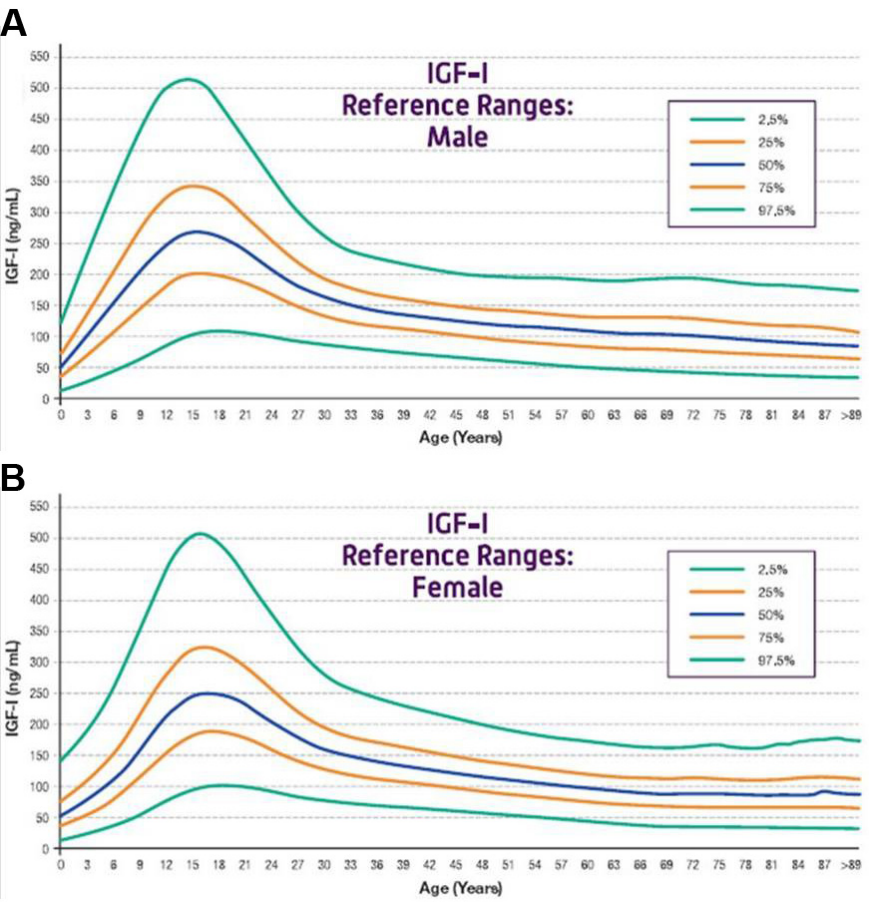
Age-adjusted reference ranges for the IDS iSYS insulin-like growth factor (IGF)-I assay. For both males (**A**) and females (**B**), there is a peak in serum IGF-I levels during puberty and a gradual decline during adulthood.

The serum creatinine was assayed using high-performance liquid chromatography, which had been standardized using isotope dilution mass spectroscopy. Hemoglobin A1c levels were also measured using high-performance liquid chromatography. The serum IGF-I levels were measured from serum samples using an automated competitive immunoassay that was calibrated to the new World Health Organization standard. This assay does not cross-react with insulin, proinsulin, or IGF-II, and shows no interference with the high-affinity insulin-like growth factor binding proteins [14]. Figure 1 shows the sex- and age-adjusted normal data for this assay [15]. The Emory University laboratory is certified by the Clinical Laboratory Improvement Amendments (CLIA).

### Statistical analysis

Data collected in the medical history questionnaire included demographic variables such as age, sex, height, weight, and race (self-assigned as white, black, or Asian). The presence of hypertension or macrovascular disease was also recorded. Subjects were considered to have macrovascular disease if they had ever been diagnosed with a myocardial infarction or a cerebrovascular accident, or if they had undergone coronary artery-bypass grafting surgery or a cardiac stenting procedure. Additionally, the duration of diabetes (defined as the interval between a subject’s diagnosis of type 2 diabetes and the time of enrollment in this study) and insulin usage were recorded for each diabetic patient. The estimated glomerular filtration rate (GFR) was calculated according to the Modification of Diet in Renal Disease formula [16].

The means and percentages for patient characteristics were compared across study groups using one-way ANOVA and the χ^2^ test, respectively. Comparisons of mean IGF-I levels were made across categorical patient characteristics using one-way ANOVA and two sample *t*-tests. For comparing the mean IGF-I across race, the six Asian patients were excluded because of the small sample size. Correlation coefficients were calculated to determine associations between IGF-I concentrations and continuous patient characteristics. Tukey’s multiple comparison procedure was used to test pairwise comparisons of patient characteristics that were significantly different across groups and to test pairwise comparisons of mean IGF-I concentrations that were significantly different across patient characteristics. Statistical calculations were performed using statistical analysis system (SAS version 9.2; Cary, NC), and statistical significance was set at a two sided p value of 0.05.

## Results

### Subject characteristics

Table 1 shows the clinical characteristics of the four study groups. There was no statistical difference between the groups with regard to age, race, or sex. The mean body mass index (BMI) was statistically lower for the no DM group than the PDR group. Additionally, the mean hemoglobin A1c was significantly lower in the no DM group than in the other three groups. The mean serum creatinine was significantly lower in the no DM and no BDR groups, compared to the NPDR and PDR groups. The mean estimated GFR was significantly higher in the no DM and no BDR groups, compared to the NPDR and PDR groups. The percentage of subjects with macrovascular disease was significantly lower in the no DM group, compared to the NPDR and PDR groups. The percentage with hypertension was statistically lower in the no DM and no BDR groups, compared to the NPDR and PDR groups.

**Table 1:** Clinical characteristics of the four study groups.

**Clinical characteristic**	**No DM (n=99)**	**No BDR (n=42)**	**NPDR (n=41)**	**PDR (n=43)**	**p-value**
Age (years)*	61.5±12.6	62.4±11.2	68.4±9.9	59.8±11.8	0.708
Race: Black	48 (49%)	21 (50%)	22 (54%)	23 (54%)	0.964
White	49 (49%)	19 (45%)	18 (44%)	19 (44%)	
Asian	2 (2%)	2 (5%)	1 (2%)	1 (2%)	
Sex: Male	49 (49%)	22 (52%)	22 (54%)	22 (51%)	0.971
Body Mass Index* (kg/m^2^)	28.4±7.1	31.7±9.6	31.3±6.6	33.0±6.7	<0.001
Duration of Diabetes (years)*	-	7.5±7.7	19.2±11.1	22.2±10.5	<0.001
Hemoglobin A1c*	5.8±0.4	7.5±1.9	7.4±1.2	8.1±1.8	<0.001
Insulin Usage	-	10 (24%)	30 (73%)	31 (72%)	<0.001
Macrovascular Disease	13 (13%)	6 (14%)	14 (33%)	19 (44%)	<0.001
Hypertension	56 (57%)	28 (67%)	39 (95%)	40 (93%)	<0.001
Serum Creatinine* (mg/dl)	0.99±0.64	0.92±0.28	1.90±1.85	2.50±2.78	<0.001
Estimated GFR* (ml/min/1.73m^2^)	82.22±24.01	89.00±29.76	58.32±31.78	47.70±29.87	<0.001

*Values indicate the mean±standard deviation. Abbreviations: DM represents diabetes mellitus, BDR represents background diabetic retinopathy, NPDR represents non-proliferative diabetic retinopathy, GFR represents glomerular filtration rate.

### Insulin-like growth factor I analysis

**Figure 2:**
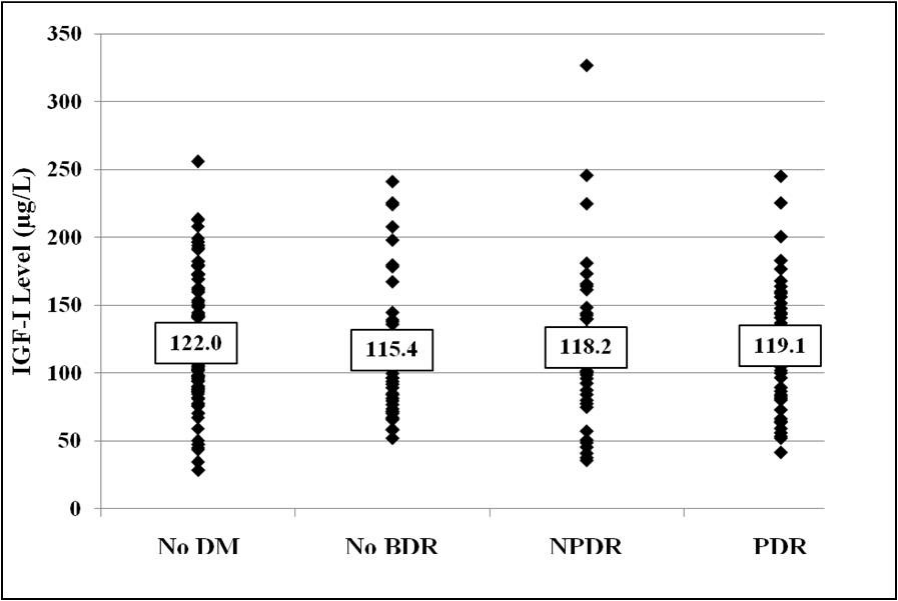
Serum insulin-like growth factor (IGF)-I concentrations for individual patients. Between the four study groups, there was no significant difference in serum IGF-I levels (p=0.897). The numerical value listed for each group represents the mean serum IGF-I concentration.

Diabetic subjects had a similar mean serum IGF-I concentration compared to nondiabetic subjects (117.6±52 µg/l versus 122.0±44 µg/l; p=0.497). The mean serum IGF-I concentrations for the groups were as follows: no DM=122.0±44 µg/l, no BDR=115.4±50 µg/l, NPDR=118.3±58 µg/l, PDR=119.1±48 µg/l. There was no significant difference in mean serum IGF-I levels among the study groups (p=0.897; Figure 2). Among the diabetic groups, the mean serum IGF-I concentration was similar among insulin-dependent and non-insulin-dependent subjects (116.8±47 µg/l versus 118.2±55 µg/l; p=0.876).

**Figure 3:**
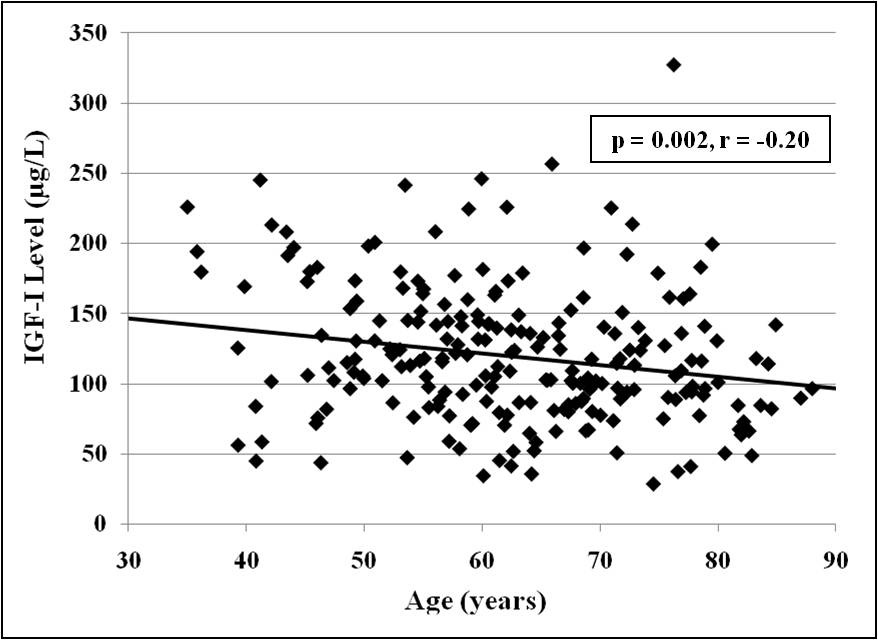
Serum insulin-like growth factor (IGF)-I concentration in relation to age. When all study subjects were considered, there was a statistically significant inverse correlation between serum IGF-I concentration and age.

There was no statistically significant difference in mean serum IGF-I concentrations according to sex (p=0.174), macrovascular disease (p=0.375), or hypertension (p=0.131). Additionally, there was no statistically significant association between IGF-I levels and hemoglobin A1c (p=0.430, r=−0.05), serum creatinine (p=0.181, r=0.09), or estimated GFR (p=0.369, r=−0.06). While not significantly different, there was a trend toward decreasing serum IGF-I concentrations with increasing duration of diabetes (p=0.111, r=-0.14). There was a statistically significant association between IGF-I and age (p=0.002, r=−0.20), and between IGF-I and body mass index (p=0.008, r=−0.18). Figure 3 shows that IGF-I levels gradually declined with increasing age. There was a significant difference in mean IGF-I concentrations between racial groups (p=0.040). In a subanalysis of race, from which Asian subjects were excluded, black subjects had higher IGF-I levels than did white subjects (126.9±47 µg/l versus 112.9±49 µg/l; p=0.03).

## Discussion

The purpose of this cross-sectional study was to assess the relationship between serum IGF-I levels and diabetic retinopathy using a novel immunoassay that was calibrated against an international standard. This study found no association between serum IGF-I levels and diabetic retinopathy. Additionally, there were no differences in serum IGF-I levels among insulin-dependent diabetic subjects and non-insulin-dependent subjects. Congruent with other studies, serum IGF-I levels were statistically lower in older subjects.

The role that IGF-I plays in diabetic retinopathy remains somewhat controversial. Poulsen first suggested a possible relationship between growth hormone or IGF-I and diabetic retinopathy after he noted the regression of proliferative diabetic retinopathy following pituitary infarction [17]. This relationship was further supported after experimental studies showed that pituitary ablation resulted in the regression of diabetic retinopathy [2–4]. Merimee found that growth hormone-deficient dwarfs with diabetes exhibited no microvascular complications [18], and later showed that serum IGF-I levels in adult diabetic patients with rapidly progressive retinopathy were twice those of patients without retinopathy, of patients with less severe retinopathy, and of nondiabetic control patients [7]. Later clinical studies by Sato et al. and Dills et al. also found higher levels of serum IGF-I in patients with PDR [8,9]. Interestingly, this study showed no correlation between diabetic retinopathy and serum IGF-I levels. The variation between these studies’ findings may be a result of the assays used to measure IGF-I levels.

The advances in IGF-I assay methodology is critical when considering the accuracy of previous trials. Most modern IGF-I assays have a low cross-reactivity for IGF-II and binding proteins [19]. However, the problems of many of the assays include a lack of age-adjusted normal data, lack of standardization, interference from binding proteins, and lack of a pure international reference population [19]. These problems have been minimized by the IDS iSYS assay, which is a robust IGF-I assay that has been calibrated against a pure international standard and age-adjusted normative data [14]. Furthermore, the assay does not cross-react with either insulin, pro-insulin, or IGF-II, and shows no interference from the IGF-binding proteins. This assay provides a sensitive and specific interpretation of serum IGF-I, which is a strength of the current study.

While this study found no association between serum IGF-I levels and diabetic retinopathy, it is still possible that local IGF-I plays a role in the development of diabetic retinopathy. Experimental evidence suggests that increased the intraocular concentration and activity of IGF-I may be responsible for microvascular changes in diabetic retinopathy. Several studies have shown that elevated intraocular levels of IGF-I, IGF-II, and IGF-binding protein-3 correlate with the degree of retinal ischemia in diabetic patients [20,21]. Spranger et al. [22] also showed that intravitreal levels of IGF-I were elevated in patients with PDR and that this elevation correlated with plasma levels. They suggested that the increased intraocular IGF-I concentrations were a result of the spillover of serum protein, but could not rule out the possibility of local production. Furthermore, they showed that retinal photocoagulation did not influence intraocular levels of IGF-I [23]. Guidry and colleagues [24] showed that vitreous fluid from patients with diabetic retinopathy possessed an increased capacity to stimulate the tractional force generated by Muller cells because of the increased activity of IGF-I, IGF-II, and platelet-derived growth factor. In another study, Simo et al. [25] found that both vascular endothelial growth factor and free IGF-I levels were elevated in diabetic patients with PDR, compared to nondiabetic controls. However, because vitreous levels of IGF-I did not correlate with active PDR status, they concluded that vascular endothelial growth factor was directly involved with the pathogenesis of PDR, whereas the precise role of IGF-I remained to be established.

Serum IGF-I levels have been related to age in the general population, with low levels in childhood, peak levels during puberty, and gradually declining levels in adulthood. While the current study only focused on adult subjects with type 2 diabetes, there was a significant difference in IGF-I levels according to age. This finding correlates well with that of Tan and Baxter [26], who showed a decline in serum IGF-I levels with increasing age in an adult-onset diabetic population. Nardelli and colleagues [27] found no correlation between IGF-I and age in their insulin-using and non-insulin-using diabetic groups, but did find a negative correlation between IGF-I and age in their nondiabetic control population. The association between IGF-I and age in the current study should not change according to insulin status, because the IGF-I levels were virtually identical in the insulin-dependent and non-insulin-dependent diabetic subjects (116.8 µg/l versus 118.2 µg/l).

Many of the subjects in this study, particularly those with diabetic retinopathy, had concomitant systemic hypertension. It should be noted that the blood pressure measurements of these patients were not recorded. Instead, patients were asked whether they had been previously diagnosed with hypertension. Interestingly, there was no significant association between serum IGF-I concentrations and the presence of hypertension (p=0.131). This finding contrasts previous studies that show that lower levels of serum IGF-I correlate with systemic hypertension [28–30]. Furthermore, IGF-I has been implicated in the development of hypertension through its inotropic and growth-promoting effects on the heart and endothelium [31]. Possibly due to patient characteristics or small sample sizes, we did not find an association between serum IGF-I concentrations and hypertension. It is also possible that an association between serum IGF-I and hypertension could have been found if we had collected blood pressure measurements. Further studies are needed to clarify the pathogenic role IGF-I has in the development of hypertension.

As the kidney is a major site of IGF-I degradation, it could be expected that IGF-I levels may be higher in patients with renal insufficiency [32]. Hyer and colleagues [11] found this to be true. Interestingly, the current study found no association between serum creatinine levels or estimated GFR and IGF-I levels. This result is similar to other studies that found no association between IGF-I and renal failure [8,10,12]. It is unclear why these studies were unable to find an association between IGF-I and renal insufficiency. It is possible that an association exists, but it is weak.

There were a few limitations to the current study. First, its cross-sectional design limited our ability to assess causality. Second, only one time point was recorded for the subjects. A longitudinal analysis of these patients, with serial fundoscopic examinations and blood testing, would be valuable for determining whether our findings hold true over time. Furthermore, only serum IGF-I levels were measured. As mentioned earlier, the serum IGF-I assay we used did not cross-react with IGF-II, and it minimized the interference from the high-affinity binding proteins. It thus should have reflected the true serum IGF-I level. Nonetheless, it might have been useful to measure IGF-II levels and IGF-binding proteins to see if there were any associations with the IGF-I levels or with diabetic retinopathy.

In conclusion, this large cross-sectional study found no association between serum IGF-I levels and diabetic retinopathy. Our results call into question whether serum IGF-I contributes to the pathogenesis of diabetic retinopathy. Additional prospective studies are needed to assess the potential significance of IGF-I as a risk factor for diabetic retinopathy, and also to evaluate the effects of suppressing systemic or intraocular IGF-I on the progression of retinopathy.
